# Reciprocal interactions between circadian clocks and aging

**DOI:** 10.1007/s00335-016-9639-6

**Published:** 2016-05-02

**Authors:** Gareth Banks, Patrick M. Nolan, Stuart N. Peirson

**Affiliations:** MRC Harwell, Harwell Science and Innovation Campus, Oxfordshire, OX11 0RD UK; Sleep and Circadian Neuroscience Institute (SCNi), Nuffield Department of Clinical Neurosciences, Oxford Molecular Pathology Institute, Sir William Dunn School of Pathology, South Parks Road, Oxfordshire, OX1 3RE UK

## Abstract

Virtually, all biological processes in the body are modulated by an internal circadian clock which optimizes physiological and behavioral performance according to the changing demands of the external 24-h world. This circadian clock undergoes a number of age-related changes, at both the physiological and molecular levels. While these changes have been considered to be part of the normal aging process, there is increasing evidence that disruptions to the circadian system can substantially impact upon aging and these impacts will have clear health implications. Here we review the current data of how both the physiological and core molecular clocks change with age and how feedback from external cues may modulate the aging of the circadian system.

## Aging and the Aged Society

Aging is characterized by a gradual decline in numerous physiological systems. These widespread changes include a reduction in metabolic function (Krems et al. 2005), an increase in fat mass and reduction in lean mass (Atlantis et al. 2008), disrupted sleep/wake cycles (Dijk et al. 1999; Huang et al. 2002), a decline in immune function leading to increased susceptibility to disease (Haynes and Maue 2009), reduced cognitive performance (Samson and Barnes 2013), and a decline in retinal function (Gao and Hollyfield 1992). Advances in modern medicine and health care have increased life expectancy world-wide and while this is obviously beneficial at the level of the individual, it brings into sharp focus how the changes associated with aging will impact upon society (World Health Organization 2002). Like all biological processes, aging is influenced by both genetic factors and environmental factors and therefore a more complete understanding of the complex interplay between these factors is of primary importance in our knowledge of the aging process. While the effect of environmental factors such as diet is widely acknowledged (e.g., Ingram et al. 2001), the influences of daily light cycles and internal biological (circadian) cycles are often overlooked in discussions of aging.

## The Mammalian Circadian System

**Fig. 1:**
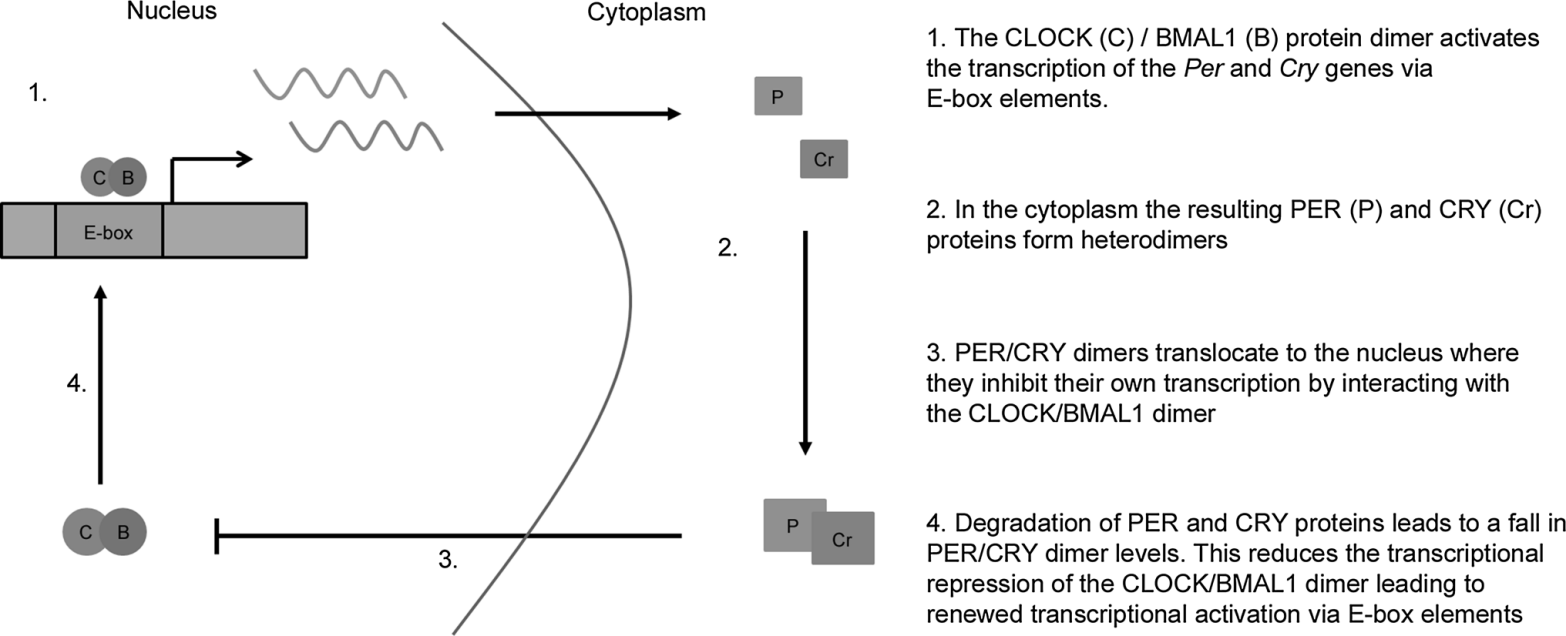
Simplified representation of the mammalian molecular clock. The core clock genes constitute a transcriptional feedback loop which maintains a period of approximately 24 h. Note that CLOCK and BMAL1 regulate the expression of two *Per* genes (*Per1* and *Per2*) and two *Cry* genes (*Cry1* and *Cry2*). A more detailed review of the molecular clock can be found in Mohawk et al. 2012

Throughout the body, a vast range of biological processes are modulated by an internal circadian clock (from the Latin ‘around a day’). There is a measureable circadian component to processes such as sleep, cognition, cardiac function, digestion, hormone synthesis and secretion, body temperature, and gene expression (Huang et al. 2011; Kwon et al. 2011; Yu and Weaver 2011). The suprachiasmatic nucleus (SCN) of the hypothalamus acts as a master circadian pacemaker, regulating other (peripheral) clocks throughout the body and thus maintaining physiological rhythmicity. The core clock mechanism which underpins these rhythmic changes is a cell autonomous intracellular transcriptional–translational feedback loop (TTFL) which oscillates with a period of approximately 24 h (Fig. 1). The TTFL itself comprises a number of ‘clock genes’ [including period 1 and 2 (*Per1*-*2*), cryptochrome 1 and 2 (*Cry1*-*2*), clock (*Clock*), and Bmal1 (*Bmal1* or *Arntl*)] and the transcription of various downstream genes is controlled either via transcriptional action of core clock genes (Kondratov et al. 2006a, b) or via clock output genes such as *Dbp* (Gachon et al. 2006). The time and tissue specificity of these downstream ‘clock-controlled’ genes regulates the peripheral clocks found throughout the body. It is also notable that many clock-controlled genes are themselves transcription factors which will drive secondary transcriptional rhythms within cells, allowing the generation of rhythms not directly regulated by the core clock genes. Expression analysis has demonstrated that the rhythms generated by different transcription factors have different properties (for example regulation by DBP elements will drive high-amplitude rhythms compared to those driven by E-boxes) (Ueda et al. 2005). Additionally, post-translational mechanisms such as RNA interference (Wang et al. 2015) and protein ubiquitination (Godinho et al. 2007) can further regulate the cycling of the core clock genes, the clock-controlled genes, and their corresponding outputs. These multiple levels of regulation allow for great complexity and flexibility in both the core and peripheral clocks found throughout the body.

In the absence of external cues, these clocks will maintain rhythmicity with a period (tau, τ) of around 24 h. However, as a key function of the circadian clock is to predict changes in the environment, the core clock needs to synchronize with (or become entrained to) external time cues (*zeitgebers*). The most important *zeitgeber* for the mammalian circadian clock is the light/dark (LD) cycle, which is detected by retinal photoreceptors, including rods, cones, and melanopsin-expressing photosensitive retinal ganglion cells (pRGCs). These light input signals are transmitted to the SCN via the retino-hypothalamic tract, where they act as phase setting signals for the master circadian clock (Hughes et al. 2015).

## Aging and Physiological Rhythms

**Fig. 2:**
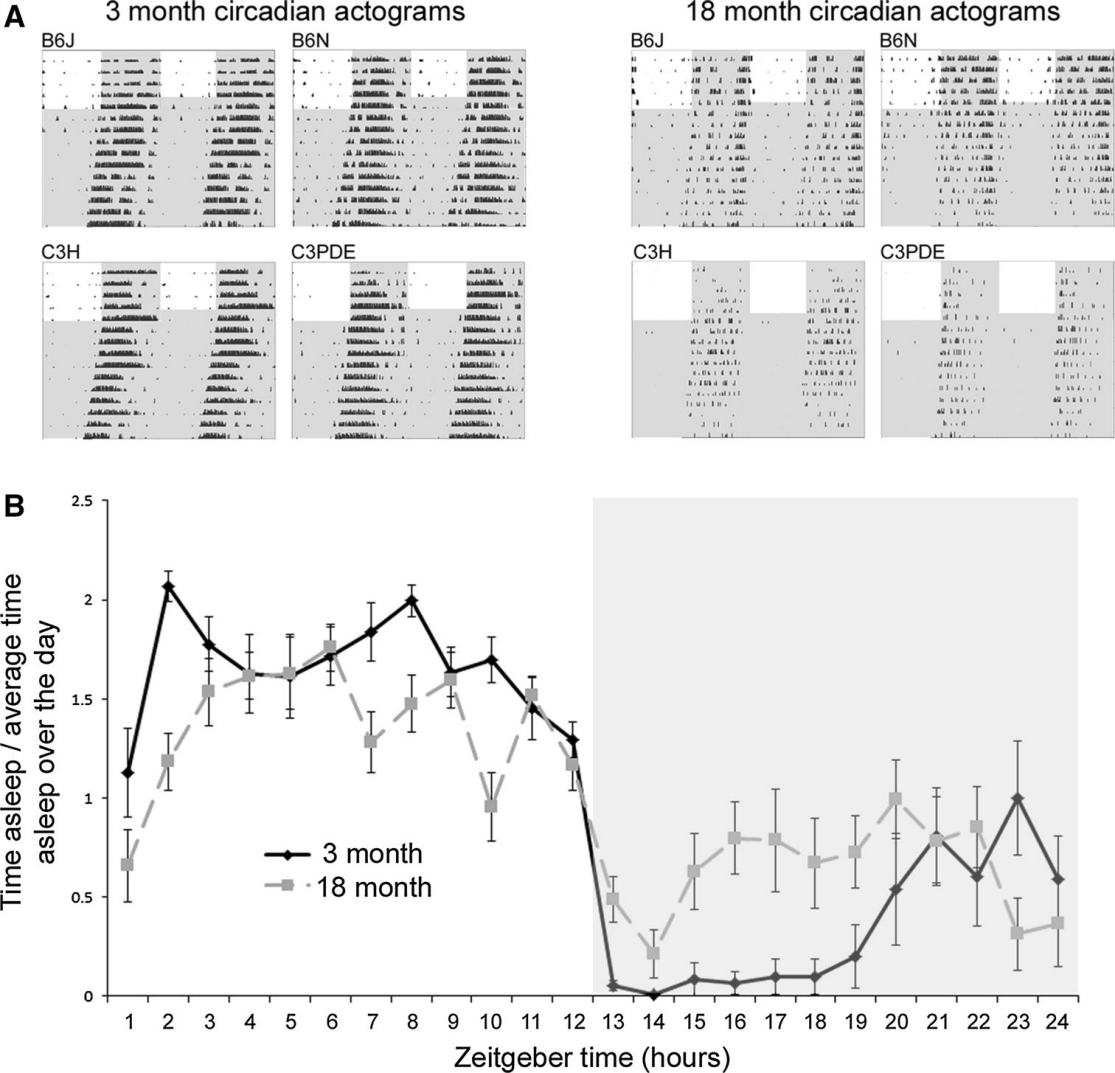
Activity profiles of young and aged mice demonstrate the breakdown of the circadian system with age. **a** Double-plotted actograms of four different mouse strains at 3 and 18 months of age. These activity profiles demonstrate that mice show a number of age-related changes including reduced activity, reduced circadian amplitude, and lengthened circadian period. Figure reproduced from Banks et al. 2015. **b** Circadian rhythm of sleep behavior in 3- and 18-month-old mice. Older mice show poor rhythmicity and reduced amplitude in their rhythms. Figure adapted from Banks et al. 2015

Throughout life, the internal circadian processes slowly deteriorate. For example, in humans, the rhythms of sleep become more fragmented with age (a phenomenon described as a loss in circadian amplitude) and the timing of the sleep phase becomes progressively earlier (Roenneberg et al. 2007; Espiritu 2008). Similarly, the amplitude and magnitude of the rhythms of eating and of hormone secretion are also reduced with age (Dijk et al. 1999; Van Cauter et al. 1998; Pandi-Perumal 2005). Although there is some evidence to the contrary (Monk et al. 1995), body temperature also appears to show an age-related reduction in circadian amplitude (Weinert 2010). Such age-related circadian changes have been observed in animal models, including a shift to earlier activity onset and disruptions to the sleep–wake cycle (Banks et al. 2015; Farajnia et al. 2012). Activity records in aged mice show clear loss of rhythmicity and circadian amplitude compared to younger animals (Fig. 2).

Evidence in mice also suggests that aged animals have a less adaptable circadian system (Azzi et al. 2014) and are more susceptible to the detrimental effects of phase shifts in the light cycle (e.g., jet-lag) than younger animals (Davidson et al. 2006). Additionally, it has been shown in mice that the ability of the core clock to respond to external cues and stimuli is diminished with age (Biello 2009; Lupi et al. 2012).

## Aging and the Suprachiasmatic Nucleus

**Fig. 3:**
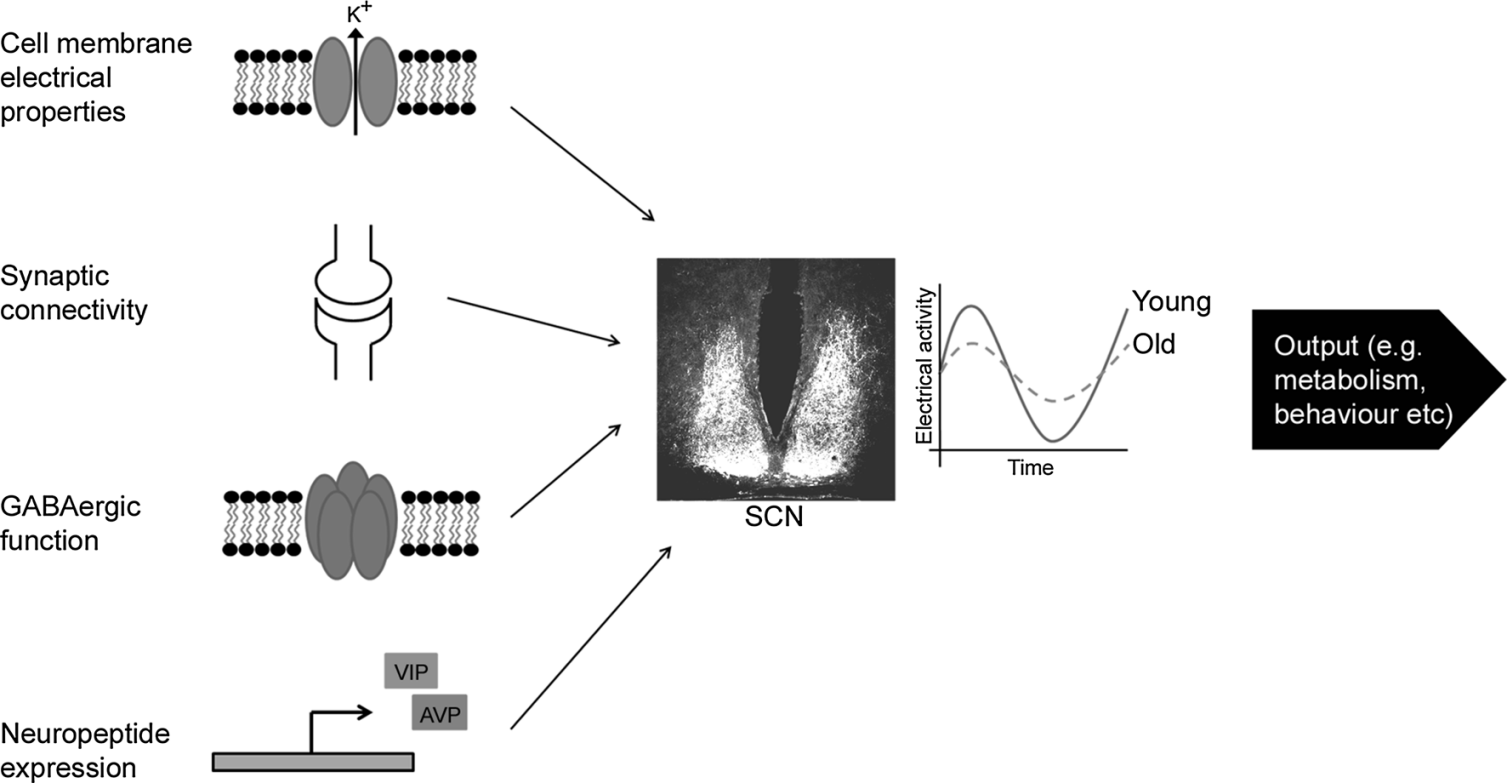
Loss of SCN electrical rhythms is due to the contribution of disruptions to various components of intra-SCN communication. Age-related changes in the electrical membrane properties, synaptic connectivity, GABAergic function, and expression of neuropeptides of SCN neurons all contribute to a loss of synchronization of the SCN network. This is reflected by an age-related loss of the amplitude of electrical rhythms of the SCN

Recent studies have demonstrated that the age-related changes to circadian physiology described above are also reflected at a cellular level. As it is the site of the master circadian pacemaker, the majority of these studies have focused upon changes to the SCN and how these changes may modulate downstream circadian processes. Multiunit neuronal activity recordings of the SCN have demonstrated that there is an age-related loss of amplitude in electrical rhythms (Nakamura et al. 2011). While this reduction does not appear to be due to a reduction in the total number of cells in the SCN (Miller et al. 1989), changes in cellular properties, neuronal connectivity, and gene expression appear to underlie this decline (Fig. 3). The electrical properties of SCN neurons change with age, with older neurons showing a reduction in the circadian amplitude of resting membrane potential and potassium currents (Farajnia et al. 2012). Recent studies have suggested that these electrical changes may be the result of changes to large conductance calcium-activated potassium channels (BK channels) (Farajina et al. 2015). In addition to these changes at a single cell level, there are also age-related changes to cellular communication across the SCN. Quantification of synaptic terminals in the SCN has demonstrated an age-related reduction in synaptic spines and a shortening of dendrites, suggesting a loss of neuronal connectivity with age (Palomba et al. 2008). Additionally, there is an age-related loss of expression of the neuropeptide vasointestinal polypeptide (VIP) in the SCN (Duncan et al. 2001) and the rhythmic expression of both VIP and the neuropeptide arginine-vasopressin (AVP) shows a delay in peak expression with age (Cayetanot et al. 2005). Since neuropeptides such as VIP and AVP play important roles in synchronizing cellular rhythms within the SCN (Maywood et al. 2006), these gene expression changes will further disturb the rhythmic output of the neuronal network. Furthermore, GABAergic signaling appears to be disrupted in the aged SCN (Palomba et al. 2008; Nygard et al. 2005) and, as GABA also contributes to neuronal synchronization, this loss may further disrupt rhythmicity within the SCN. The breakdown of these four components (the electrical properties of individual cells, synaptic connectivity, neuropeptide expression, and GABAergic function) will each impact the synchronization of the neuronal network of the SCN. It therefore becomes increasingly difficult for the rhythms of individual neurons to remain synchronized with those of others in the network and thus the rhythmic output of the network becomes fragmented, reflected in a loss of amplitude in circadian processes (Fig. 3).

## Aging and Gene Expression—the Core Clock and Beyond

Given the age-related cellular and network changes within SCN neurons, it may be predicted that there will be similar age-related changes at the level of circadian gene expression. A number of studies have compared the expression of core genes that make up the molecular clock in the SCN of young and old animals (Table 1). While these studies show a consistent age-related decline in expression of the clock gene *Bmal1* and a consistent lack of age-related change in the expression of *Per1*, there is no clear consensus with regard to the other core clock genes. It is however notable that a study which used the bioluminescent reporter gene PER2::LUC to track expression of PER2 protein in aged mice suggested that housing animals in 12-h light: 12-h dark lighting cycles masked some of the age-related changes in gene expression that were revealed by aging in constant dark conditions (Nakamura et al. 2015).

**Table 1:** Age-related changes in the expression of core clock genes in the SCN

Gene	Study (model organism)	Change in total expression due to aging	Change in rhythmicity due to aging
*Per1*	Kolker et al. (2003) (Hamster)	No change	No change
	Weinert et al. (2001) (mouse)	No change	No change
	Asai et al. (2001) (rat)	No change	No change
	Bonaconsa et al. (2014) (mouse)	No change	Tendency to phase advance
*Per2*	Chang and Guarente (2013) (mouse)	↓	Not reported
	Kolker et al. (2003) (Hamster)	No change	No change
	Weinert et al. (2001) (mouse)	↓	Reduced amplitude
	Asai et al. (2001) (rat)	No change	No change
	Nakamura et al. (2011) (mouse)	No change	No effect in IHC/reduced amplitude in bioluminescence
	Bonaconsa et al. (2014) (mouse)	↓	Tendency to phase advance
*Cry1*	Weinert et al. (2001) (mouse)	No change	Not rhythmic in young or old^a^
	Asai et al. (2001) (rat)	↓	Reduced amplitude
	Bonaconsa et al. (2014) (mouse)	No change	Not rhythmic in young; becomes rhythmic in old
*Cry2*	Bonaconsa et al. (2014) (mouse)	No change	Not rhythmic in young or old
*Clock*	Kolker et al. (2003) (Hamster)	↓	Not rhythmic in young or old
	Weinert et al. (2001) (mouse)	No change	Not rhythmic in young or old
	Bonaconsa et al. (2014) (mouse)	No change	Not rhythmic in young or old
	Wyse (2010) (mouse)	↓	Not rhythmic in young; becomes rhythmic in old
*Bmal1* (*Arntl*)	Chang and Guarente (2013) (mouse)	↓	Not reported
	Kolker et al. (2003) (Hamster)	↓	No change
	Bonaconsa et al. (2014) (mouse)	↓	Rhythmic in young; arrhythmic in old
	Wyse (2010) (mouse)	↓	Not rhythmic in young or old

^a^Sampling not performed at peak or trough of cycle

The age-related changes to core clock gene expression also appear to be under direct genetic modulation. The NAD^+^-dependant protein deacetylase SIRT1 has been demonstrated to play a key role in the age-related breakdown of the core molecular clock (Chang and Guarente 2013). In aged mice, the levels of SIRT1 in the SCN are reduced suggesting an age-related decline in expression of the protein. Young *Sirt1* knockout mice show a number of phenotypes indicative of older wildtype mice (e.g., longer circadian period and reduced expression of *Per2* and *Bmal1*), whereas *Sirt1*-overexpressing mice are protected from these age-related changes (Chang and Guarente 2013). SIRT1 affects circadian rhythms by modulating the activity of the clock proteins CLOCK and BMAL1 (Nakahata et al. 2008) and the age-related changes in its expression may help account for age-related changes in clock gene expression and thus to the breakdown of circadian rhythms in later life. An additional level of age-related control of rhythmic gene expression in the SCN may be DNA methylation. Studies in which mice are housed in non-24-h light cycles demonstrate that young mice show plasticity in their circadian rhythms that older mice lack (Azzi et al. 2014). Further investigation demonstrated that this plasticity was controlled by methylation of the DNA of the SCN and that inhibiting methylation inhibited the plastic reprogramming observed in young mice. Although further investigations are required, it is plausible that such DNA methylation changes may also control the age-related changes in gene expression in the SCN.

At this juncture, it is worth noting that mice lacking the core clock genes *Clock* or *Bmal1* show a number of phenotypes consistent with premature aging (Dubrovsky et al. 2010, Kondratov et al. 2006a, b). This can be interpreted as direct evidence for a role of the core clock in modulating healthy aging. However, it has been noted that there is a lack of consistency between knockout models of the clock genes in general with regard to age-related phenotypes. For example, mice made arrhythmic through the disruption of both *Per* or both *Cry* genes do not show the same age-related phenotypes as arrhythmic mice lacking *Bmal1*, suggesting that the age-related phenotypes found in *Clock* and *Bmal1* knockout mice may be due to gene-specific effects that are independent of their circadian function (Yu and Weaver 2011). Evidence for this interpretation can be found in a recent study which used an inducible knockout strategy to ablate *Bmal1* only in adult animals. Here the authors found that several of the phenotypes found in constitutive knockouts were not present in the inducible knockouts, suggesting that some of the ‘age-related’ phenotypes previously ascribed to *Bmal1* may be due to gene-specific effects during development rather than due to the impact of circadian disruption upon the aging process (Yang et al. 2016). So, although there is compelling evidence for a circadian component to aging, care must be taken in ascribing changes in the aging process directly to circadian clock genes as these genes may have non-circadian functions.

## Circadian Misalignment, Health, and Aging

The age-related changes to the circadian system described above can be seen as the consequence of the process of normal aging. However, there is also substantial evidence that if the circadian system is challenged or disrupted, there are negative consequences for both health and aging. Circadian disruption is commonly caused by the misalignment of the internal circadian clock and the external influence of *zeitgebers*. A common example of this is jet-lag, in which rapid travel across time zones leads to a mismatch either between the external day/night cycle and the internal clock or between the core and peripheral clocks. Misalignment can also occur when our behavioral and physiological rhythms are forced out of phase with the day/night cycle due to work schedules. Such work-based misalignment includes chronic shift work and social jet-lag. Additionally, in modern societies, there is an increasing prevalence of ‘light at night’ in which light signals from artificial lighting occur at inappropriate phases of the circadian cycle. Such misalignment between the circadian system and the environment has been shown to negatively impact upon health. Exposure to light at night is associated with depression in rodents (Fonken and Nelson 2013) and has been hypothesized to associate with an elevated risk of cancer (Kloog et al. 2011) and obesity (Wyse et al. 2011) in humans. Social jet-lag has been associated with obesity, metabolic disorder, cardiovascular risk, and endocrine function (Parsons et al. 2015; Rutters et al. 2014). Chronic shift work has a number of negative impacts including reduced performance in a number of tasks and elevated risks of cardiovascular disease and cancer (Evans and Davidson 2013; Schernhammer et al. 2013). Recent evidence has demonstrated not only that this circadian misalignment prematurely ages the brain, but also that it can take years to recover from prolonged disruption of the circadian system (Marquie et al. 2015).

Circadian misalignment has been studied in animal models by exposing them to abnormal light/dark cycles. This often involves subjecting animals to a non-24-h LD cycle or ‘T-cycle’ (e.g., 11-h light, 11-h dark, or T22). Animals maintained in such environments show a number of negative consequences including obesity, learning and memory deficits, and neuronal abnormalities (Gibson et al. 2010; Karatsoreos et al. 2011). Very short T7 cycles which prevent circadian entrainment have been shown to affect mood-related behavior and cognitive function in mice—a response that appears to be mediated via melanopsin-expressing photosensitive retinal ganglion cells (LeGates et al. 2012). While such lines of evidence demonstrate a clear health risk to circadian rhythm misalignment, the circadian disruption applied in many such studies is transient, rather than applied throughout the lifespan of an organism. However, there is evidence that even mild disruption to the circadian system can be detrimental to aging when applied over long periods. Early studies using fruit flies (*Drosophila melanogaster*) or blow flies (*Phormia terraenovae*) demonstrated that their lifespan is significantly increased when they are raised in LD cycles of 24 h when compared to flies raised in cycles either longer or shorter than 24 h (Pittendrigh, and Minis 1972, von Saint Paul and Aschoff 1978). Later, studies using cyanobacteria demonstrated that, if two competing strains of are grown under the same culture conditions, the strain with an internal circadian clock which most closely matches the period of the environment will outcompete the other (Ouyang et al. 1998). Such studies have been seen as evidence for ‘circadian resonance’ which suggests that an organism’s fitness is enhanced when its internal clock matches that of the external environment, and that the greater the difference between the internal and external rhythms, the greater the damage to the fitness of an organism (Pittendrigh et al. 1958).

There is some evidence that circadian resonance may also apply in mammals. Wildtype mice with a circadian period of approximately 24 h show reduced longevity when housed under short T-cycles (Park et al. 2012). Conversely, *tau* mutant hamsters, carrying a mutation which shortens their circadian period to approximately 22 h, show reduced longevity when housed in 24-h LD cycles (Hurd and Ralph 1998). However, if these mutants are maintained either in the absence of light cycles or under a 22-h T-cycle (which matched their short period), longevity and health are unaffected (Hurd and Ralph 1998, Martino et al. 2008). In addition, using data from a number of lifespan and circadian studies, Wyse et al. (2010) demonstrated that, for both mice and primates, the deviation of the internal circadian period from 24 h was inversely proportional to average lifespan. More detailed studies have demonstrated that, in mice, deviations of 7 min or more between the internal and external cycles will significantly reduce lifespan (Libert et al. 2012). Additionally, a recent study demonstrated that, in outdoor populations of mice, a mutation in the gene casein kinase 1ε (which causes a significant shortening of circadian rhythms) was strongly selected against, further suggesting that mice with abnormal circadian periods show a reduction in fitness across lifespan (Spoelstra et al. 2016). Although it is arguable that this effect on fitness is the result of non-circadian deleterious effects of the casein kinase 1ε mutation, the authors note that a similar study found that a mutation in the core clock gene *Per2* (which, in contrast to the casein kinase 1ε mutation, has negligible effect upon circadian period) had no effect upon outdoor fitness of a population (Daan et al. 2011).

## Feedback from Aging to Rhythms and Future Directions

While the evidence discussed above shows that circadian rhythms break down with age and that the aging process itself can be modulated by misalignment of the circadian system, it is also notable that SCN activity can be directly modulated by circadian activity via neuronal feedback pathways (Hughes and Piggins 2014). It is therefore difficult to consider age-related changes affecting only one aspect (e.g., cellular properties, gene expression, etc.) of the circadian system, as each will feed back and influence the others. For example, it has been demonstrated that the presence of a running wheel in a mouse cage will alter rhythmicity at cellular, metabolic, and physiological levels (Yasumoto et al. 2015). This, coupled with the circadian resonance hypothesis outlined above, suggests a hierarchy of how circadian misalignment may affect the aging process: aging in a cycle misaligned to the internal rhythm is clearly deleterious to health (for example see Spoelstra et al. 2016); aging in the absence of an external cycle (thus removing aligning signal to the internal circadian rhythm) appears to then be healthier (for example see Hurd and Ralph 1998); finally aging under conditions in which the internal and external rhythms reinforce each other appears to be most healthy (for example see Nakamura et al. 2015). In this context, it is notable that *tau* mutant hamsters (which show poor health and reduced longevity when housed in 24-h light cycles) show improved health if the SCN (and therefore the internal rhythm) is ablated (Martino et al. 2008). From such studies, there are clear potential health benefits to understanding the impact of the circadian system upon the aging process. However, while the evidence that circadian dysfunction both impacts upon and is modulated by the aging process is compelling, several important questions remain regarding the nature of this interaction. Currently, it remains unclear whether the aging of any particular physiological systems (e.g., metabolism, cognition, immune function, etc.) is more at risk from circadian misalignment than others. Additionally, it is important to establish whether such age-related changes are reversible and how the reintroduction of ‘healthy’ rhythms may ameliorate the deleterious effects of circadian misalignment. Studies in elderly care homes have demonstrated that the use of light and melatonin as circadian synchronizers has beneficial effects on certain symptoms of dementia, suggesting that understanding the health benefits of strengthening circadian rhythms may have clinical applications (Riemersma-van der Lek et al. 2008). It is also vital to understand the cellular and genetic components of the circadian impact upon aging. A number of hypotheses have been advanced regarding how circadian dysfunction may impact at a cellular level, including increased oxidative stress (Musiek et al. 2013) and changes in telomerase activity (Chen et al. 2014), but the relationship between these processes and the circadian system in the context of aging has yet to be fully investigated. Age-related changes in the expression of circadian clock genes are likely to underpin such cellular changes. While at present a greater understanding of these changes is required to establish the relative contributions to specific genes or cellular mechanisms to the circadian process, such investigations may prove invaluable in the search for possible pharmacological targets to promote healthy aging.

Overall, the impact of circadian rhythms is clearly worthy of consideration in discussions of aging and the aging process. The challenges associated with an increasingly aged population have never been more relevant and, in this context, knowledge of the impact of the circadian system upon aging and the potential health benefits of healthy rhythms may prove both economically and sociologically invaluable.
